# Cohesive zone length of metagabbro at supershear rupture velocity

**DOI:** 10.1007/s10950-016-9588-2

**Published:** 2016-06-02

**Authors:** Eiichi Fukuyama, Shiqing Xu, Futoshi Yamashita, Kazuo Mizoguchi

**Affiliations:** 1 National Research Institute for Earth Science and Disaster Resilience, Tsukuba, Japan; 2 Central Research Institute of Electric Power Industry, Abiko, Japan

**Keywords:** Dynamic rupture propagation, Cohesive zone length, Supershear rupture, Friction experiments

## Abstract

We investigated the shear strain field ahead of a supershear rupture. The strain array data along the sliding fault surfaces were obtained during the large-scale biaxial friction experiments at the National Research Institute for Earth Science and Disaster Resilience. These friction experiments were done using a pair of meter-scale metagabbro rock specimens whose simulated fault area was 1.5 m × 0.1 m. A 2.6-MPa normal stress was applied with loading velocity of 0.1 mm/s. Near-fault strain was measured by 32 two-component semiconductor strain gauges installed at an interval of 50 mm and 10 mm off the fault and recorded at an interval of 1 MHz. Many stick-slip events were observed in the experiments. We chose ten unilateral rupture events that propagated with supershear rupture velocity without preceding foreshocks. Focusing on the rupture front, stress concentration was observed and sharp stress drop occurred immediately inside the ruptured area. The temporal variation of strain array data is converted to the spatial variation of strain assuming a constant rupture velocity. We picked up the peak strain and zero-crossing strain locations to measure the cohesive zone length. By compiling the stick-slip event data, the cohesive zone length is about 50 mm although it scattered among the events. We could not see any systematic variation at the location but some dependence on the rupture velocity. The cohesive zone length decreases as the rupture velocity increases, especially larger than $\sqrt{2}$ times the shear wave velocity. This feature is consistent with the theoretical prediction.

## Introduction

In the framework of linear elastic fracture mechanics, shear stress and slip velocity diverge at the rupture front (e.g., Kostrov 1964). In this case, the stress intensity factor characterizes the fracture at the crack tip (e.g., Freund 1990; Broberg 1999).

**Fig. 1:**
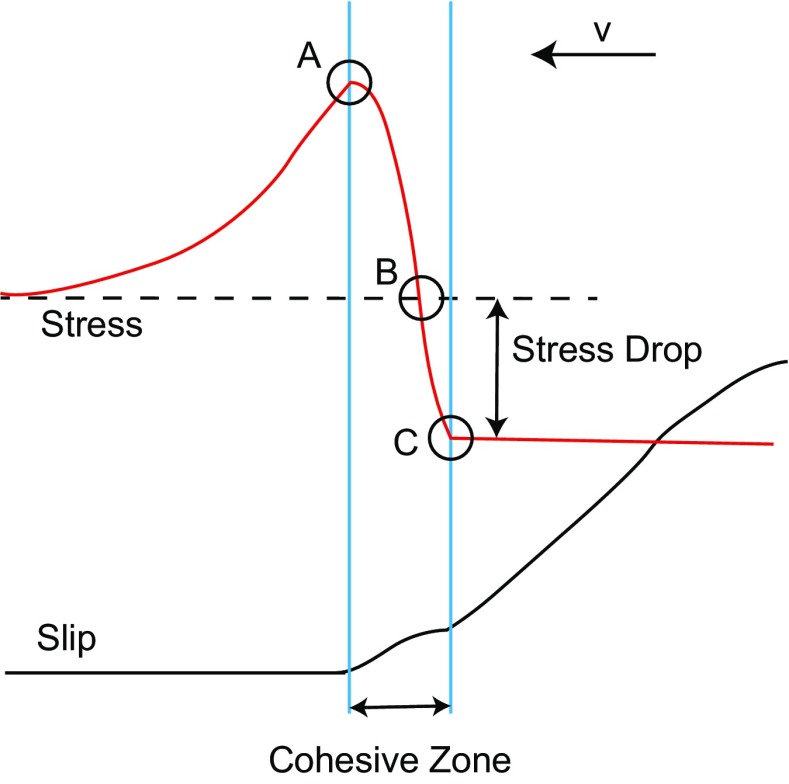
Schematic illustration of the shear stress and slip with the cohesive zone based on the theoretical formulation (Ida 1972). The onset of the cohesive zone in shear stress is the point *A* where the slip starts. And, the end of the cohesive zone is the point *C* where the shear stress reaches frictional stress level. The point *B* indicates the position where the stress becomes lower than the initial stress level

In contrast, it has been recognized that the stress concentration at the crack tip produces a cohesive zone where inelastic deformation occurred to make the stress finite (e.g., Udias et al 2014). This feature has been introduced as a slip-weakening friction where shear strength decreases as slip advances at the crack tip (Ida 1972; Ohnaka 2013 and references therein). In the framework of slip-weakening constitutive law, cohesive zone model was introduced (Fig. 1) where cohesive zone length (*l*_*c*_) is defined as a length of cohesive zone and is considered as one of the important parameters to characterize the rupture process of the earthquake (Andrews 1985).

In mode II rupture cases, a steady-state rupture propagates with either slower than the Rayleigh wave velocity (*v*_*R*_) or between shear (*v*_*S*_) and compressional (*v*_*P*_) wave velocities (Burridge 1973). It is well known that for sub-Rayleigh rupture, the cohesive zone length becomes shorter as the rupture speed becomes faster and approaches to zero at *v*_*R*_ (Broberg 1999). But, it is not clear how *l*_*c*_ behaves with rupture velocity at supershear rupture regime. It should be noted that the above feature assumes a steady-state situation which might be different from that in the present study.

There are several theoretical studies on *l*_*c*_ as a function of rupture velocity at supershear regime. However, these results are not always consistent. Kubair et al. (2002), Samudrala et al. (2002), and Bhat et al. (2007) predicted that *l*_*c*_ diverges as the rupture velocity approaches *v*_*S*_, while Huang and Gao (2001) suggested that *l*_*c*_ approaches zero at *v*_*S*_. Similarly, at *v*_*P*_, Kubair et al. (2002) and Samudrala et al. (2002) predicted that *l*_*c*_ diverges, while Huang and Gao (2001) and Bhat et al. (2007) suggested that it approaches zero at *v*_*P*_. These differences might come from different boundary conditions or assumptions made in their computations. Therefore, it should be important to investigate the behavior of *l*_*c*_ in the laboratory.

Actually, there are very few experimental studies to estimate *l*_*c*_ for rock samples although there are many studies for the mode I rupture of metals (e.g., Broberg 1999). So, it should be important to report the value of *l*_*c*_ for shear rupture on rock samples measured in the laboratory. This estimate may be very useful for the interpretation of the rupture process of the natural earthquakes.

At the National Research Institute for Earth Science and Disaster Resilience (NIED), Tsukuba, Japan, a series of friction experiments were conducted using a meter-sized rock specimens on the shaking table facility (Fukuyama et al. 2014; Yamashita et al. 2015), where mode II rupture dominated. By using a meter-sized rock specimen, *l*_*c*_ values become measurable in the laboratory. In this paper, we employed the data during the large-scale friction experiments with gabbroic rock specimens to observe the detailed behavior of the rupture propagation of stick slip events. We focus on the cohesive zone length when rupture propagates faster than *v*_*S*_ (i.e., supershear rupture).

## Experiments

**Fig. 2:**
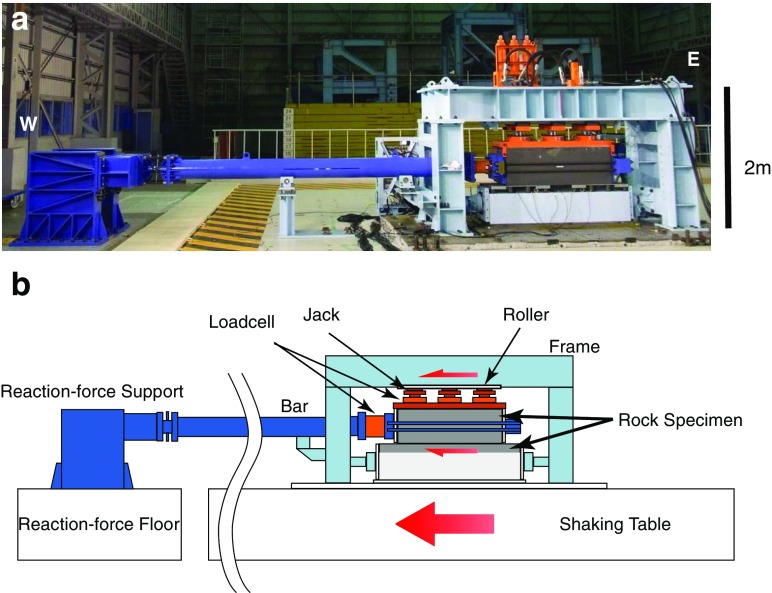
**a** A photograph of the apparatus. The *scale* is shown in the *right side* of the photo. *Left* and *right sides* correspond to west and east, respectively, and the photo is taken from the south. **b** A schematic illustration of the apparatus. *Red arrows* indicate the moving direction

Several large-scale friction experiments were constructed at NIED, on the shaking table whose dimension is 15 m by 14.5 m and maximum displacement is 0.44 m. The shaking table motion was used to apply a loading force to the rock specimens. In Fig. 2, a photograph of the apparatus with its schematic illustration is shown. The yellow-black hatched zone is the boundary of the shaking table, which moves toward west (left) in the picture during the experiment. The reaction force support, on the other hand, tries to prevent the upper specimen from moving with the shaking table, while the lower specimen is fixed to the shaking table. Then, relative displacement takes place between upper and lower specimens.

We used a pair of meter-scaled rock specimens, and the nominal fault area was 1.5 m in length and 0.1 m in width. The rock is metagabbro from Tamil Nadu, south India. Material properties of the rock are shown in Table 1. Regarding the elastic wave velocities, *v*_*P*_ and *v*_*S*_, we have two estimates. One is estimated based on the Young modulus, Poisson ratio, and density, and the other is estimated directly using high-frequency acoustic waves, both of which are shown in Table 1. Young modulus and Poisson ratio were evaluated by uniaxial compression testing by the rock company. Density was also provided by the rock company (Sekigahara Co. Ltd., personal communication). *v*_*S*_ value is more or less consistent with the two estimates, but *v*_*P*_ is quite different. In the present study, we use the values estimated by the mechanical constants because the rupture properties are more related to that estimate.

**Table 1:** Rock specimens used for the experiments

Item	Value
Rock type	Metagabbro
Locality	Tamil Nadu, south India
Major composition	Plagioclase, pyroxene, hornblende, quartz, and magnetite
Average grain size	∼1 mm
Young modulus	103 GPa
Poisson ratio	0.31
Density	2980 kg/m^3^
P-wave velocity	6919 m/s^a^
(*v*_*p*_)	6560 m/s^b^
S-wave velocity	3631 m/s^a^
(*v*_*S*_)	3700 m/s^b^

^a^Estimated by Young modulus, Poisson ratio, and density

^b^Estimated by acoustic wave measurements

To make a perfect contact between the fault surfaces, we prepared a very flat sliding surface whose undulation is less than 10 μm following the Japanese Industrial Standard (JIS) B7513 (http://www.jisc.go.jp), which corresponds to International Organization for Standardization (ISO) 8512-2 (http://www.iso.org). This is the initial condition of the fault surface for the first experiment. We used the same set of rock specimens repeatedly after removing, each time, the gouge produced by the previous experiment. Experiments were done under room temperature and room humidity condition.

In each experiment, we applied a normal stress of 2.6 MPa to the rock specimens. Then, we started loading at a constant velocity of 0.1 mm/s until the total slip reached 40 mm. Macroscopic normal and shear stresses were measured by the load cells shown in Fig. 2. Since three jacks were used to apply normal stress, three load cells were used to measure the total normal force (see Fig. 2). In contrast, shear force was measured by the load cell attached horizontally between the upper rock specimen and the reaction force support bar. The coefficient of friction is measured as the ratio of shear force to the normal force.

**Fig. 3:**
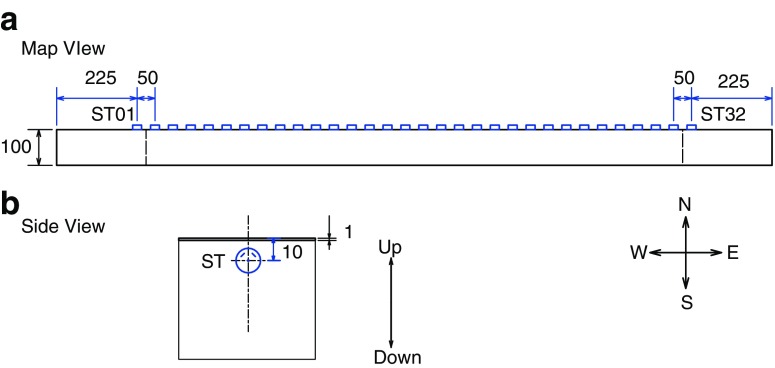
Locations of the strain gauges. **a** Map view of their locations. *Blue open squares* indicate the locations of the sensors. Orientation of the rock specimen is defined with respect to north, south, east, and west. **b** The side view of the strain gauge sensor. The specimen is tapered 1 mm at the edge, and the sensor is installed 10 mm lower than the slip surface. Two orthogonal strain gauge components are oriented 45° from the slip surface as shown in th*e blue line* in the *blue circle*. The unit of the numerals is in millimeters

Thirty-two two-component semiconductor strain gauges (KSN-2-120-F3-11, Kyowa Inc.) were installed at an interval of 50 mm along the edge of sliding surface 10 mm off the fault (Fig. 3). Two gauge components are orthogonal, and both oriented 45° from the slip surface. Signals were conditioned by high-frequency (up to 0.5 MHz) strain amplifier (CDA-900A, Kyowa Inc.), and local shear strain is recorded continuously at a sampling interval of 1 MHz with 16-bit resolution (Spectrum Inc. M2i.4741).

## Analysis

**Fig. 4:**
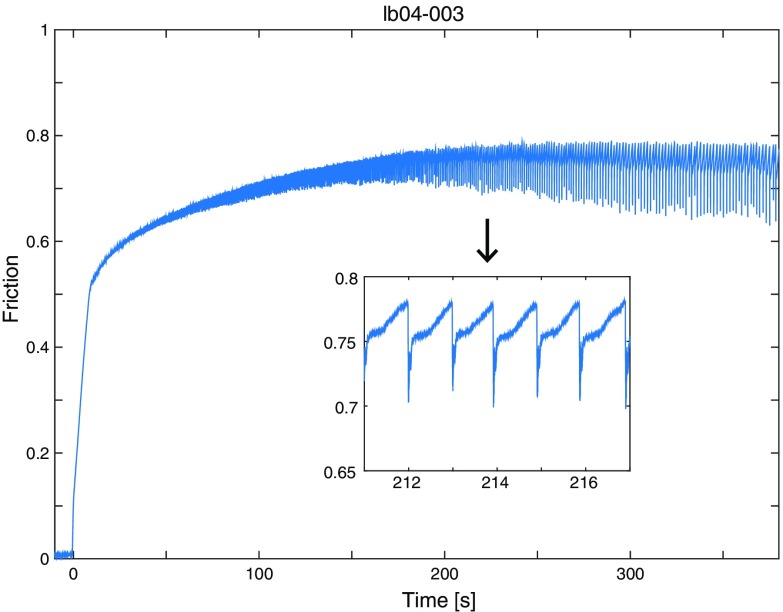
Friction curve as a function of time for experiment LB04-003. *Inset* is a magnified plot between 211 and 217 s. One can see that the stick slips occurred continuously

In Fig. 4, temporal variation of coefficient of friction during an experiment is shown. In the inset of Fig. 4, which is a magnified plot between 211 and 217 s, we can see many stick slip events whose stress drop is about 0.1 in coefficient of friction (or about 0.3 MPa in shear stress drop). We analyzed the shear strain array data at each stick slip event. It should be noted that for the analysis of the *l*_*c*_ estimation, we used the data of the third experiment in LB04 specimen series, called “LB04-003.” The reason why we chose this data set is simply because there are many supershear rupture events included. Actually, it was quite difficult to control the rupture velocity of the stick slip events because the propagation velocity depends on the fault surface roughness as well as the amount of off-fault damages, which we could not control yet.

Figure 1 shows a schematic illustration of stress and slip behavior at the rupture front based on the theoretical formulation. This will help for the explanation of the method to estimate *l*_*c*_ here. Cohesive zone is defined as the distance between the peak stress and residual stress along the fault at the rupture front. This zone is considered as inelastic behavior so that the stress does not diverge at the rupture front and gradually decreases behind the rupture front toward the frictional stress level. If we have a spatial variation of shear strain along the fault as shown in Fig. 1, we will be able to measure the *l*_*c*_ value by picking the position for the peak strain (A in Fig. 1) and that for the residual strain (C in Fig. 1). However, since the strain gauge was installed slightly off the sliding surface, the strain waveforms were contaminated by elastic waves. This makes the detection of the residual strain location (C in Fig. 1) practically difficult. Thus, we picked the position where the shear strain drops to the initial strain value (B in Fig. 1) instead of picking the residual position. Therefore, in this paper, we call the distance between A and B the cohesive zone length (*l*_*c*_). It should be reminded that this *l*_*c*_ value is an approximation to directly measure from the observation data. We will discuss the accuracy of this approximation later using theoretical strain waveforms.

It should be noted, however, that what we measured is the temporal variation of shear strain, which can be converted to shear stress by multiplying the rigidity of the medium. And, what we need to estimate *l*_*c*_ is the spatial variation of shear stress. Since we could measure the propagation velocity of the rupture front, we are able to estimate the spatial distribution of shear strain by converting the spatial array of temporal strain change. When the rupture velocity (*v*) is constant, *x-vt* becomes the variable of the array data, where *x* and *t* are spatial and temporal coordinate parameters, respectively. Therefore, the temporal strain data *s*(*x*_0_, *t*) at position *x*_0_ can be considered as a spatial snapshot of *s*(*vt*_0_ *, -x/v*) at time *t*_0_ if *t∼t*_0_ and *x∼x*_0_.

**Fig. 5:**
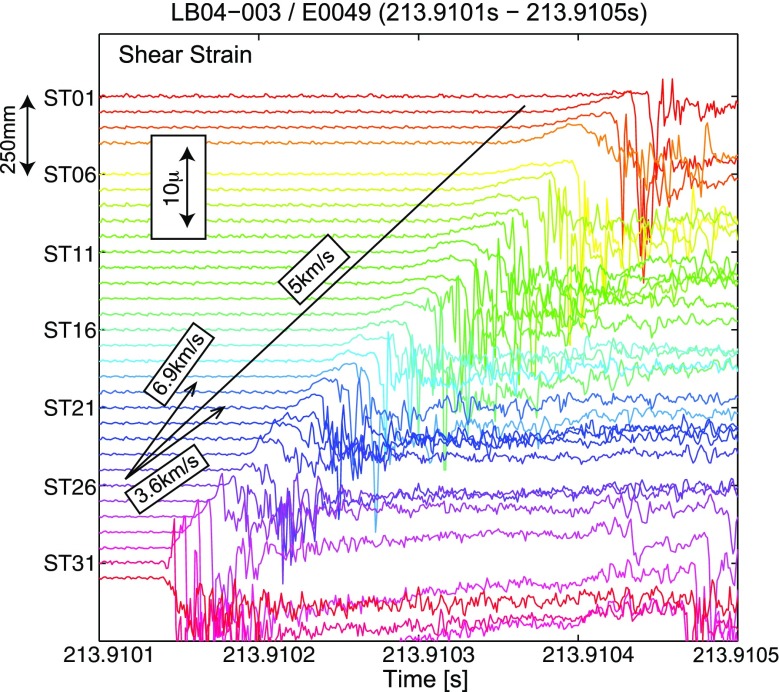
An example of observed strain waveforms for the event E0049 that occurred during the experiment of LB04-003 during 213.9101 and 213.9105 s. The sensor locations are shown in *left axis*, and the *scale* of the amplitudes is shown as an *inset*. As a reference, the rupture velocity of 5 km/s is shown as a *solid line*

In Fig. 5, 32 shear strain waveforms are shown for the stick slip event E0049, as an example of the observed data. This event was initiated between ST31 and ST32 at 213.91015 s after the loading started and the rupture propagated mostly unilaterally at a velocity of 5.0 km/s. We estimated *v* by a least square fitting of the rupture time data. The rupture time was measured by picking the time when the strain waveform suddenly decreased and crossed the initial strain level before the stick slip (B in Fig. 1). The estimated *v* is much faster than the *v*_*s*_ of this material (3.63 km/s) and is close to $\sqrt{2}{v}_s$.

**Fig. 6:**
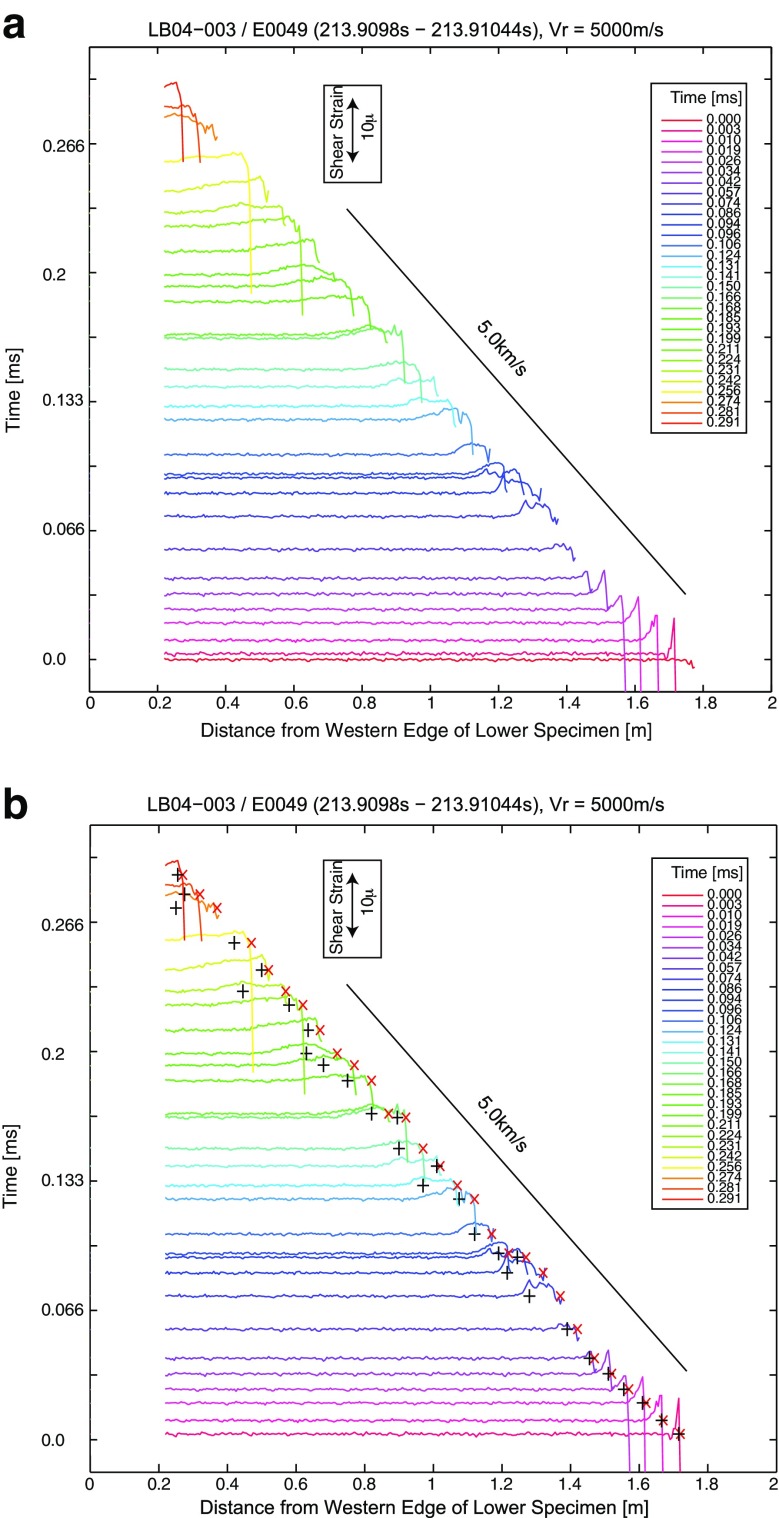
**a** Converted shear strain distribution along the fault at specific times for Event E0049 of the experiment LB04-003. The time is shown in the *left axis*, and the *scale* for the strain amplitudes is shown in the *inset*. In **b**, the same plot is shown with peak strain locations (*black pluses*) and rupture front locations (*red crosses*)

In Fig. 6, snapshots of the spatial distribution of the strain ahead of and around the rupture front for the stick slip event E0049 are plotted, which were converted from the temporal strain array data along the fault (Fig. 5) assuming a constant *v* of 5.0 km/s. In this plot, we assume that the reference time for the space-time conversion is set at the rupture front arrivals at an observation point. Then, we consider the data recorded before this time as the data recorded in space ahead of the rupture propagation at this reference time by adjusting the space-time coordinate using the assumed rupture velocity. In Appendix A, we show the comparison between converted spatial strain distribution with the snapshot of the strain along the fault, which was the measured strain value at a particular time at each position. We can see that the conversion works reasonably well. Using these plots, we estimated *l*_*c*_ values by measuring the distance between A and B in Fig. 1.

It should be noted that, as can be seen in Fig. 6b, at some temporal snapshots, the peak strain location was not clear, which could be due to the local rupture velocity perturbation or due to the station location at finite distance off the fault. The local velocity variation might be related to heterogeneous stress drop along the fault. And, when the observation point is not on the fault, near-fault waves might contaminate the strain waveforms. Since we did not take into account these effects here, these could result in estimation errors of *l*_*c*_ values in the present study.

## Results

In the experiment of LB04-003, we detected 187 stick slip events. Among them, we selected ten stick slip events without any preceding foreshocks (Table 2, for details). If foreshocks occur ahead of the mainshock, the mainshock rupture front is contaminated by the stress and strength change caused by the occurrence of the foreshocks. Then, we could not apply a simple model that the rupture front propagates at constant velocity. It should be noted that these events initiated at similar locations close to ST31 (see Fig. 3). Therefore, the *l*_*c*_ value estimated at a different location corresponds to a different propagation distance.

**Table 2:** Details of stick slip events

Event name	Origin time (s)	*v* ^a^ (m/s)	Std (*v*)^b^ (m/s)	Mean *l*_*c*_ (m)
E0049	248.40010	4946	39	0.045
E0057	257.94545	4250	130	0.043
E0058	259.08130	4645	91	0.054
E0102	303.79350	5124	93	0.025
E0137	356.70940	5082	84	0.039
E0139	361.29940	5259	92	0.043
E0142	367.52050	5397	101	0.036
E0147	377.68790	5337	75	0.031
E0156	393.16560	5004	96	0.024
E0159	397.73875	5283	71	0.032

^a^Rupture velocity

^b^Standard deviation of rupture velocity.

**Fig. 7:**
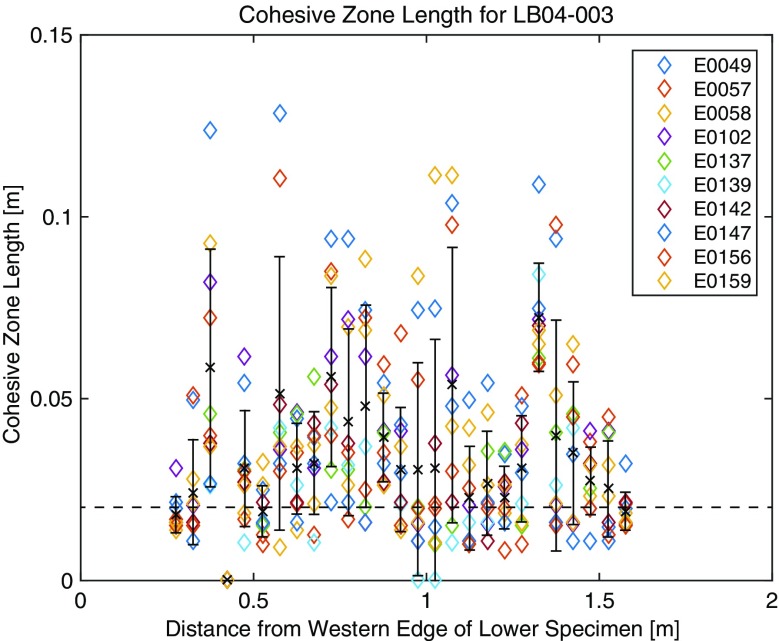
The measured cohesive zone length plotted as a function of the location. *Different colors* correspond to different stick slip events. *Crosses* and *bars* stand for the average values and their standard deviations. *Broken horizontal line* indicates the minimum resolvable *l*_*c*_ values (0.02 m) that will be shown in Fig. 8b

In Fig. 7, we show *l*_*c*_ values measured at each location on the fault for ten stick slip events. The mean values and their standard deviations of *l*_*c*_ are plotted as crosses and bars, respectively. Since *l*_*c*_ values are scattered with large standard deviations, we could not say that *l*_*c*_ is either a local parameter that is only determined by the local condition at each location or depending on the propagation distance. However, from Fig. 7, we can roughly say that *l*_*c*_ is of the order of 50 mm in this experiment. Since there are very few reports about the *l*_*c*_ value for natural rock specimens, this value should be important and could be a reference value for the supershear rupture velocity on the metagabbro under a normal stress of 2.6 MPa. It should be noted that the resolution of *l*_*c*_ depends on the sampling interval of the strain data (i.e., 1 MHz). For the propagation velocity of 5.0 km/s, the resolution of *l*_*c*_ becomes 0.005 m, which is sufficiently fine to resolve the estimated value (∼0.05 m).

**Fig. 8:**
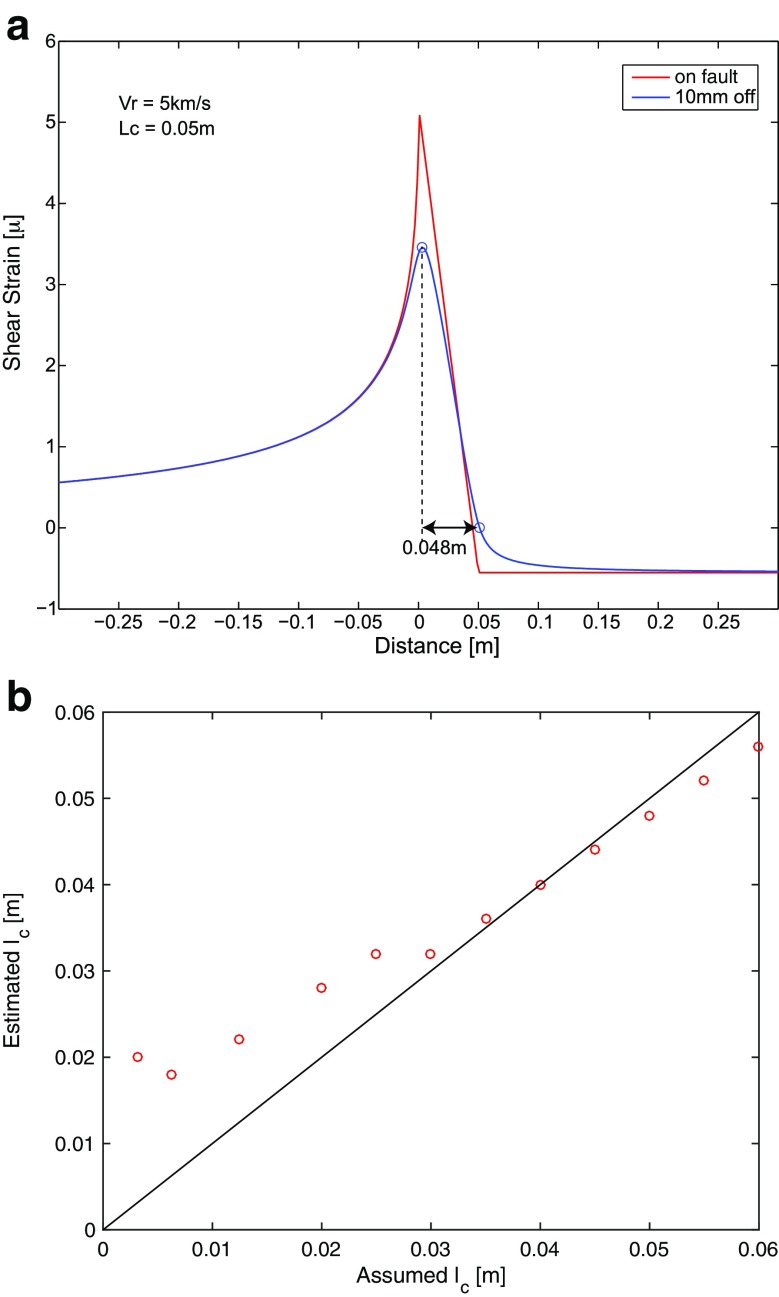
**a** Theoretical computation of the shear strain distribution along the fault. *Red line* is the assumed strain distribution on the fault, and *blue one* is the computed strain distribution 10 mm off the fault. *Two open circles* on the *blue line* indicate the peak (corresponding to *A* in Fig. 1) and zero-crossing (*B* in Fig. 1) strain locations, respectively. **b** The relations between assumed *l*_*c*_ values and estimated *l*_*c*_ values are plotted as *red circles. Solid line* is the reference for the true solution

We need to confirm how well the estimations of *l*_*c*_ are done in this study because the strain is measured not on the fault but 10 mm off the fault. If *l*_*c*_ is not large enough compared to the distance from the fault surface, we might not be able to approximate that the observation is done on the fault. To investigate whether we can consider that the observation is on the fault surface, we conducted numerical simulations. We used the numerical computation code by Bhat et al. (2007), which is based on the slip pulse model proposed by Rice et al. (2005) and extended for the supershear rupture case by Dunham and Archuleta (2005). In Fig. 8a, we show a shear strain behavior on the fault as well as 10 mm off the fault when the rupture propagates at a velocity of 5.0 km/s with *l*_*c*_ = 0.05 m. As shown in the figure, if we measure the A-B distance in Fig. 1, *l*_*c*_ is estimated at 0.048 m from the strain data 10 mm off the fault. Therefore, we think that, under the present configuration of the measurements, we could reasonably estimate *l*_*c*_ values from the strain array data installed 10 mm off the fault. In Fig. 8b, we changed the *l*_*c*_ values, computed the strain field 10 mm off the fault, and measured the *l*_*c*_ values the same way as we did. As can be seen in the figure, *l*_*c*_ values are well estimated when the real *l*_*c*_ value is greater than 0.03 m. If *l*_*c*_ is smaller than 0.01 m, estimated value cannot be greater than 0.02 m from this theoretical test. This indicates that the estimation is reliable if the estimated *l*_*c*_ value is larger than 0.03 mm. Therefore, we could say that our estimated *l*_*c*_ values have enough resolution and accuracy.

**Fig. 9:**
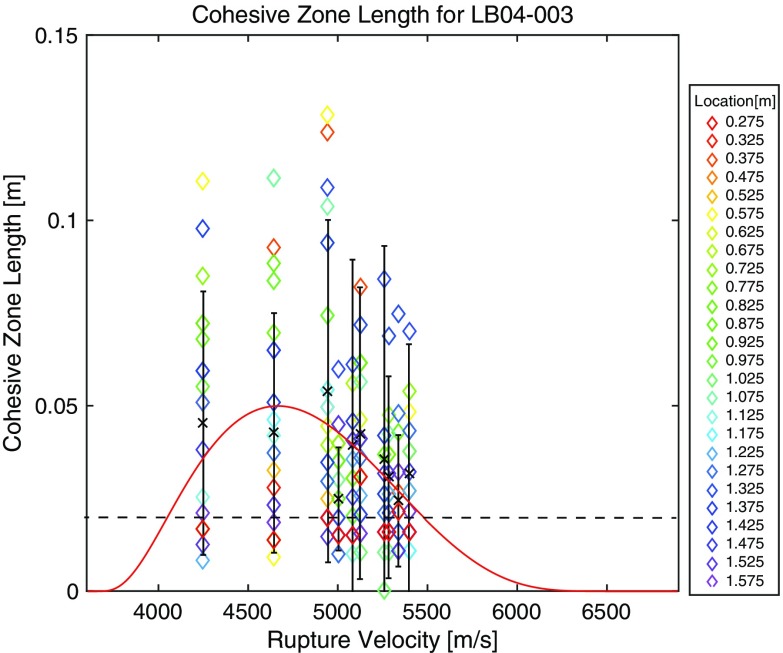
Cohesive zone lengths are plotted as a function of rupture velocity for the experiment of LB04-003. *Different colors* correspond to different locations on the fault. *Broken horizontal line* indicates the minimum resolvable *l*_*c*_ values (0.02 m) shown in Fig. 8b. *Red curve* shows the theoretical prediction based on Huang and Gao (2001)’s model

In Fig. 9, *l*_*c*_ is plotted as a function of *v*. Although there is some scattering among the stick slip events, we could see a tendency that *l*_*c*_ decreases when *v* becomes faster than 5.0 km/s, which corresponds to $\sqrt{2}{v}_s$. There are several models to explain the dependency of *l*_*c*_ on *v*. Huang and Gao (2001) derived a theoretical solution for a crack propagating with a constant rupture velocity with mixed boundary conditions where shear stress drops constant amount inside the crack and shear dislocation is zero outside the crack (see Appendix B for details). They assumed a Dugdale-Barenblatt-type cohesive zone model in the theoretical solution to estimate the *l*_*c*_ variation as a function of *v*. In their computation, *l*_*c*_ becomes the largest between *v*_*S*_ and $\sqrt{2}{v}_s$. Our observation is consistent with their theoretical prediction. And, this feature suggests that *l*_*c*_ is *v* dependent in supershear regime as theoretically predicted with the assumption of Dugdale-Barenblatt-type cohesive zone model, which is a sort of slip-weakening friction.

## Conclusion

We conduct large-scale friction experiments and obtained experimentally the cohesive zone length (*l*_*c*_) of metagabbro when rupture propagates with supershear velocity. We found that *l*_*c*_ is about 50 mm for the rupture propagation distance of less than 1.5 m. We could not observe that *l*_*c*_ is dependent on the position (i.e., rupture propagation distance). But, we observed a dependence on the rupture propagation velocity. This dependence is consistent with theoretical prediction of Huang and Gao (2001).

## Figures and Tables

**Fig. 10:**
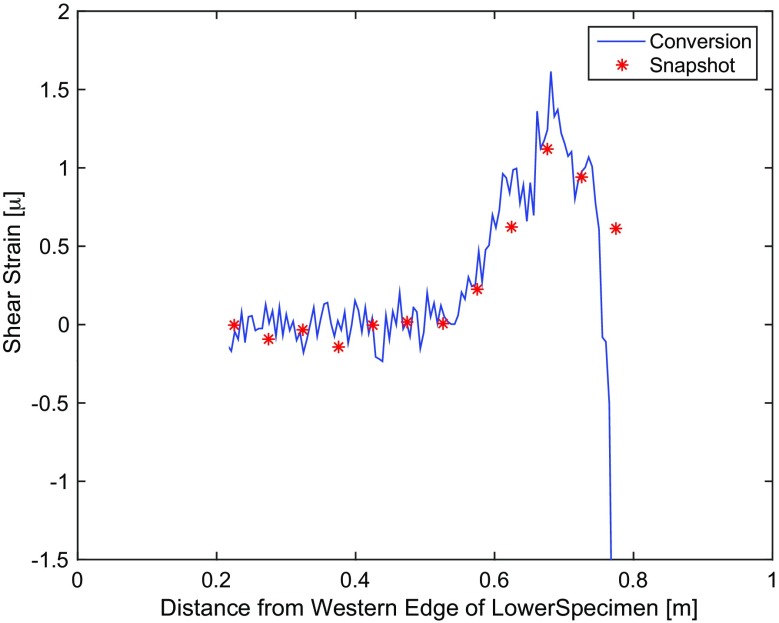
Comparison of converted spatial strain distribution at t=0.193ms (blue line) with the snapshot of the data obtained at each location along the fault at t=0.193ms (red asterisks)

## Appendix A. Validation of space-time conversion

In Fig. 10, we show a comparison between the spatial shear strain distribution converted from a temporal shear strain data (line) and snapshots of the shear strain values along the fault surface at particular time (asterisks). The converted strain data is the same as that at 0.193 ms in Fig. 6. Although the spatial interval of strain gauge was sparse, we could see that these data are consistent with the converted strain data obtained from the strain gauge at different position. This comparison indicates that the space-time conversion works reasonably.

## Appendix B. Theoretical formulation of *l*_*c*_ by Huang and Gao

Here, we show the theoretical formulation for *l*_*c*_ derived by Huang and Gao (2001) for the convenience of the readers as follows.

B1 $$ {l}_c={\left[q\sqrt{{\left(1-{\widehat{\alpha}}_S^2\right)}^4+16{\alpha}_P^2{\widehat{\alpha}}_S^2}\frac{v_S^4}{\sqrt{v_P{v}^3}\left({v}^2-{v}_R^2\right)}\frac{\tau^{\ast }}{\tau_cvt}\frac{s_{+}(0)}{s_{+}\left(1/v\right)}f(v)\right]}^{\raisebox{1ex}{$1$}\!\left/ \!\raisebox{-1ex}{$q$}\right.}\frac{v^2-{v}_S^2}{v_P+v}t $$

where

B2 $$ {\alpha}_P=\sqrt{1-\frac{v^2}{v_P^2}} $$

B3 $$ {\widehat{\alpha}}_S=\sqrt{\frac{v^2}{v_S^2}-1} $$

B4 $$ q=\frac{1}{\pi}\mathrm{t}\mathrm{a}{\mathrm{n}}^{-1}\frac{4{\alpha}_P{\widehat{\alpha}}_S}{{\left(2-\frac{v^2}{v{}_S{}^2}\right)}^2} $$

B5 $$ \frac{S_{+}(0)}{S_{+}\left(\zeta \right)}= \exp \left[\frac{\zeta }{\pi }{\displaystyle {\int}_{\raisebox{1ex}{$1$}\!\left/ \!\raisebox{-1ex}{$\left({v}_p-v\right)$}\right.}^{\infty}\mathrm{t}\mathrm{a}{\mathrm{n}}^{-1}V+(r)\frac{dr}{r\left(r+\zeta \right)}}\right] $$

B6 $$ f(v)= \exp \left[{\displaystyle {\int}_{\raisebox{1ex}{$1$}\!\left/ \!\raisebox{-1ex}{$\left({v}_P+v\right)$}\right.}^{\raisebox{1ex}{$1$}\!\left/ \!\raisebox{-1ex}{$\left(v+{v}_S\right)$}\right.}\frac{f_0(r)}{\pi r}}dr-{\displaystyle {\int}_{\raisebox{1ex}{$1$}\!\left/ \!\raisebox{-1ex}{$\left(v-{v}_S\right)$}\right.}^{+\infty}\frac{f_0(r)}{\pi r}}dr\right] $$

and

B7 $$ V+(r)=\frac{{\left(2{r}^2-\frac{v^2}{v_S^2}{\left(r+\frac{1}{v}\right)}^2\right)}^2}{4{\alpha}_P{\widehat{\alpha}}_S{r}^2\sqrt{r-\frac{1}{v_P-v}}\sqrt{r+\frac{1}{v_P+v}}\sqrt{\left|r+\frac{1}{v-{v}_S}\right|}\sqrt{\left|r+\frac{1}{v+{v}_S}\right|}} $$

B8 $$ {f}_0(r)=\mathrm{t}\mathrm{a}{\mathrm{n}}^{-1}\left[4{\alpha}_P{\widehat{\alpha}}_S\frac{f_1(r)-{\left(2-\frac{v^2}{v_S^2}\right)}^2}{{\left(2-\frac{v^2}{v_S^2}\right)}^2{f}_1(r)+16{\alpha}_P^2{\widehat{\alpha}}_S^2}\right] $$

B9 $$ {f}_1(r) = \frac{{\left[2{r}^2-\frac{v^2}{v_S^2}{\left(r-\frac{1}{v}\right)}^2\right]}^2}{r^2\sqrt{\left(r+\frac{1}{v_P-v}\right)\left(r-\frac{1}{v_P+v}\right)\left|r-\frac{1}{v-{v}_S}\right|\left|r-\frac{1}{v+{v}_S}\right|}} $$

and *τ* ^***^ and *τ*_*c*_ are constants and can be scaled by *τ*_*c*_ *vt*/*τ* ^***^.

## References

[CR1] Andrews DJ (1985). Dynamic plane-strain shear rupture with a slip-weakening friction law calculated by a boundary integral method. Bull Seismol Soc Am.

[CR2] Bhat HS, Dmowska R, King GCP, Klinger Y, Rice JR (2007). Off-fault damage patterns due to supershear ruptures with application to the 2001 Mw 8.1 Kokoxili (Kunlun) Tibet earthquake. J Geophys Res.

[CR3] Broberg KB (1999). Cracks and fracture.

[CR4] Burridge R (1973). Admissible speeds for plane-strain self-similar shear cracks with friction but lacking cohesion. Geophys J Roy Astr Soc.

[CR5] Dunham EM, Archuleta RJ (2005). Near-source ground motion from steady state dynamic rupture pulses. Geophys Res Lett.

[CR6] Freund LB (1990). Dynamic fracture mechanics.

[CR7] Fukuyama E, Mizoguchi K, Yamashita F, Togo T, Kawakata H, Yoshimitsu N, Shimamoto T, Mikoshiba T, Sato M, Minowa C, Kanezawa T, Kurokawa H, Sato T (2014). Large-scale biaxial friction experiments using a NIED large-scale shaking table. Rep Nat’l Res Inst Earth Sci Disas Prev.

[CR8] Huang Y, Gao H (2001). Intersonic crack propagation—part I: the fundamental solution. J Appl Mech.

[CR9] Ida Y (1972). Cohesive force across the tip of a longitudinal-shear crack and Griffith’s specific surface energy. J Geophys Res.

[CR10] Kostrov BV (1964). Self-similar problems of propagation of shear cracks. Appl Math Mech (PMM).

[CR11] Kubair DV, Geubelle PH, Huang YY (2002). Intersonic crack propagation in homogeneous media under shear-dominated loading: theoretical analysis. J Mech Phys Solid.

[CR12] Ohnaka M (2013). The physics of rock failure and earthquakes.

[CR13] Rice RJ, Sammis CG, Parsons R (2005). Off-fault secondary failure induced by a dynamic slip pulse. Bull Seismol Soc Am.

[CR14] Samudrala O, Huang Y, Rosakis RJ (2002) Subsonic and intersonic shear rupture of weak planes with a velocity weakening cohesive zone. J Geophys Res 107(B8):2170. doi:10.1029/2001JB000460

[CR15] Udias A, Madariaga R, Buforn E (2014). Source mechanics of earthquakes: theory and practice.

[CR16] Yamashita F, Fukuyama E, Mizoguchi K, Takizawa S, Xu S, Kawakata H (2015). Scale dependency of rock friction at high work rate. Nature.

